# Adolescent and adult risk-taking in virtual social contexts

**DOI:** 10.3389/fpsyg.2014.01476

**Published:** 2014-12-18

**Authors:** Anneke D. M. Haddad, Freya Harrison, Thomas Norman, Jennifer Y.F. Lau

**Affiliations:** ^1^The Calleva Research Centre for Evolution and Human Sciences, Magdalen College, University of OxfordOxford, UK; ^2^Department of Experimental Psychology, University of OxfordOxford, UK; ^3^Department of Zoology, University of OxfordOxford, UK; ^4^Department of Economics, University of OxfordOxford, UK

**Keywords:** adolescent, adult, decision-making, risk-taking, peer influence, social conformity

## Abstract

There is a paucity of experimental data addressing how peers influence adolescent risk-taking. Here, we examined peer effects on risky decision-making in adults and adolescents using a virtual social context that enabled experimental control over the peer “interactions.” 40 adolescents (age 11–18) and 28 adults (age 20–38) completed a risk-taking (Wheel of Fortune) task under four conditions: in private; while being observed by (fictitious) peers; and after receiving ‘risky’ or ‘safe’ advice from the peers. For high-risk gambles (but not medium-risk or even gambles), adolescents made more risky decisions under peer observation than adults. Adolescents, but not adults, tended to resist ‘safe’ advice for high-risk gambles. Although both groups tended to follow ‘risky’ advice for high-risk gambles, adults did so more than adolescents. These findings highlight the importance of distinguishing between the effects of peer *observation* and peer *advice* on risky decision-making.

## INTRODUCTION

Adolescence involves dramatic physical, social, and cognitive changes. One change which attracts considerable attention from families, the media, and policy makers is the tendency to engage in risky behavior: adolescents are more prone than adults to drive recklessly, abuse drugs and alcohol, and engage in antisocial or risky sexual behavior. Adolescent risk-taking may be driven by greater sensation-seeking and reward sensitivity at this age (Casey et al., 2008; Forbes and Dahl, 2010) coupled with the relative immaturity of ‘cognitive control’ systems implicated in the regulation of inappropriate actions. At a neural level, these differences may reflect adolescent-specific differences in striatal and nucleus accumbens regions which subserve anticipation of reward and reward prediction error (Bjork et al., 2004; Galvan et al., 2006; Cohen et al., 2010) together with prolonged structural and functional maturation of prefrontal regions associated with cognitive control (Gogtay et al., 2004; Casey et al., 2008). But what provides the ‘triggers’ for the increased tendency to take risks? One suggestion is the growth of peer influence.

Indeed, adolescents also experience major transformations in their social environment, with a growing salience of peers and their judgments (Nelson et al., 2005). Compared with children, adolescents spend more time with peers, and show increased sensitivity to peer acceptance and/or rejection (Sebastian et al., 2010). This can contribute to an array of emotional difficulties including depression, anxiety, and deliberate self-harm. But increasing peer salience may also bring increasingly sophisticated understanding of others’ emotions, beliefs, and intentions (Blakemore, 2008) which in turn, may shape the tendency for adolescents’ attitudes and behavior to become more similar to their friends’ over time (Brechwald and Prinstein, 2011). Indeed a wide range of antisocial or health-risk behavior such as alcohol use, smoking, aggressive and illegal behavior, may be susceptible to this process of peer socialization (Brechwald and Prinstein, 2011).

Is there evidence that peers can affect risk-taking behavior? In adults, the so-called ‘risky shift’ or ‘group polarization’ phenomenon is well established: group decisions are riskier than those made privately (Stoner, 1968; Moscovici and Zavalloni, 1969; Isenberg, 1986). This shift may be more exaggerated in adolescents compared to undergraduates (Hensley, 1977). Moreover, studies using self-report measures about hypothetical social scenarios have shown that peers can influence adolescents’ behaviors (Hensley, 1977; Berndt, 1979; Brown et al., 1986; Cohen and Prinstein, 2006) but these data are susceptible to a number of problems, including social desirability effects and difficulties with accuracy of retrospective memory, and/or mismatches between self-reported hypothetical and actual behavior. Observational methods of real-world risk-taking may overcome these difficulties while retaining high ecological validity. For example, observational studies of drivers show that young people tend to drive more riskily than others, but that this effect is moderated by the presence of passengers (McKenna et al., 1998; Simons-Morton et al., 2005). While valuable, these data too have important limitations; effects may be due to mere *presence* of passengers or due to specific passenger behavior (e.g., distracting the driver or speaking up to actively discourage risky behavior). More generally, such naturalistic studies do not allow participants to be allocated to different conditions that directly compare these accounts. Moreover, naturalistic studies are less standardized than experimental studies.

Others have found a compromise between self-report and observational methods by using laboratory tasks to examine peer influence on actual (rather than hypothetical) risk-taking behavior in adolescents, but under experimental conditions where subtler aspects of peer influence can be manipulated. Gardner and Steinberg (2005) used a computerized risky driving game where participants had to decide how far to continue driving a car after an orange light appeared. Stopping sooner resulted in fewer points than if the car progressed further, but at some point the light would turn red, resulting in a crash and the loss of all accumulated points for that round. Results from adolescents (age 13–16), youths (age 18–22) and adults (age 24+) showed that risk-taking behavior (i.e., driving through the orange light) decreased with chronological age; moreover participants playing alone took fewer risks than those playing with three peers present. Crucially, however, the shift toward more risky behavior in the presence of peers was exaggerated for adolescents and youths compared with adults. Behavioral results from a subsequent imaging study showed a similar trend (Chein et al., 2011). These studies go some way toward addressing the paucity of data examining how peers change actual risk-taking behavior in adolescents. However, they too have limitations: because interactions with peers were unconstrained, different participants may have received very different advice; moreover, participants may have been influenced by knowledge or assumptions about their friends’ values or orientation toward risk – or even past experiences of risk-taking with those friends. As these effects are not measured, they could confound results if not matched between age-groups.

Although much previous work has been done with real or simulated driving, some literature has investigated observer effects on gambling and similar tasks, but mostly in young adult (student) populations (Hardoon and Derevensky, 2001; Noval and Mitchell, 2003; Yechiam et al., 2008; Mishra et al., 2010). The general finding is peers can change risk-taking behavior, but again in many studies, it is unclear whether these effects were due to the mere presence of peers (e.g., in the room doing something else), due to peers’ active observation of the participants, or because the participants tended to conform to (or perhaps to resist) peers’ advice. As with research on risky driving, the distinction between effects of peer *observation* and of peer *advice* has not been previously made clear. Disentangling these effects is important for our scientific understanding of the role and influence of peers on behavior: that peer observation may not have as large an effect compared to peer advice suggests that peers do not simply play a passive role by their presence – but in fact can actively influence others. Presumably, this is linked with changes in the social brain, particularly those regions involved in social cognition, or the understanding of others’ mental states. From an applied perspective, the distinction between peer observation and peer advice could have important implications for interventions designed to reduce risk-taking behavior. For example, campaigns targeted at adolescents sometimes use peer educators or pictures of teens and youth language to convey their message more effectively (e.g., Rickert et al., 1991). If peer presence/observation alone increases risk-taking behavior, then this may not be effective. On the other hand, if teens actually follow peer advice, then using peer educators to convey “safe” advice might be a useful strategy.

In this study we examined and compared effects of *observation* by peers and of *receiving advice* from peers on risky choices on a probabilistic decision-making task among adolescents and adults during high-risk gambles. As the effect of peers is likely to be dependent on other variables such as participant/peer gender, and the nature of the relationship and any shared past events, we used a ‘virtual’ social environment whereby participants played with unknown but same age, same gender (fictitious) peers. The salience of the social context was emphasized by informing participants that the peers would be making judgments about ‘what sort of person you are’ on the basis of their game choices. Given evidence for a greater ‘risky shift’ in adolescents compared to young adults (Hensley, 1977), we expected that, adolescents would make more risky decisions when being merely observed by peers than adults. However, due to the paucity of prior data on how adults and adolescents respond to specific advice, it was less clear what to expect for the advice conditions. We tentatively suggested that, due to their increased sensitivity to reward (Casey et al., 2008; Forbes and Dahl, 2010) – in this context, winning points – adolescents would be *more* likely than adults to follow risky advice but *less* likely than adults to follow safe advice (in other words, to choose the risky option more than adults in both advice conditions, due to their motivation to gain more points). On the other hand, some evidence shows that in some contexts, adolescents may be motivated toward anticonformity to indicate their individuality and indifference to popular opinion (Nelson et al., 2009; Bodner et al., 2011; Brechwald and Prinstein, 2011); we thought this may result in adolescent participants being less likely than adults to follow risky *and* safe advice. The decision-making task included three risk levels (high-risk, medium-risk, and even gambles). However, given previous work showing differential behavior in adolescents under risky conditions, we focused on the high-risk gambles with the medium-risk and even gambles serving as comparison conditions as well as ensuring that the task was varied enough to be interesting for participants.

## MATERIALS AND METHODS

### PARTICIPANTS

40 adolescents (20 females; aged 11–18; *M* = 14.50, SD = 2.21) and 28 adults (14 females; aged 20–38; *M* = 25.07, SD = 4.65) took part. Data from four adults was subsequently excluded as they did not believe the deception (see below). Adult participants were recruited from the local community via advertising and word-of-mouth. We endeavored to limit the number of psychology undergraduates who took part because of concerns that they may have been less likely to believe that they were interacting with real peers; only one participant had studied psychology at degree level. Adolescent participants were recruited from a secondary school. Participants over 16 provided written informed consent; participants under 16 provided written informed assent and a parent provided written informed consent. The study was approved by the local ethics committee. Participants were paid for taking part [£10 for adults; £ 9 for participants in school years 10–13 (aged 14–18); £ 7 for participants in school years 7–9 (aged 11–14)].

### PROCEDURE AND MATERIALS

**FIGURE 1:**
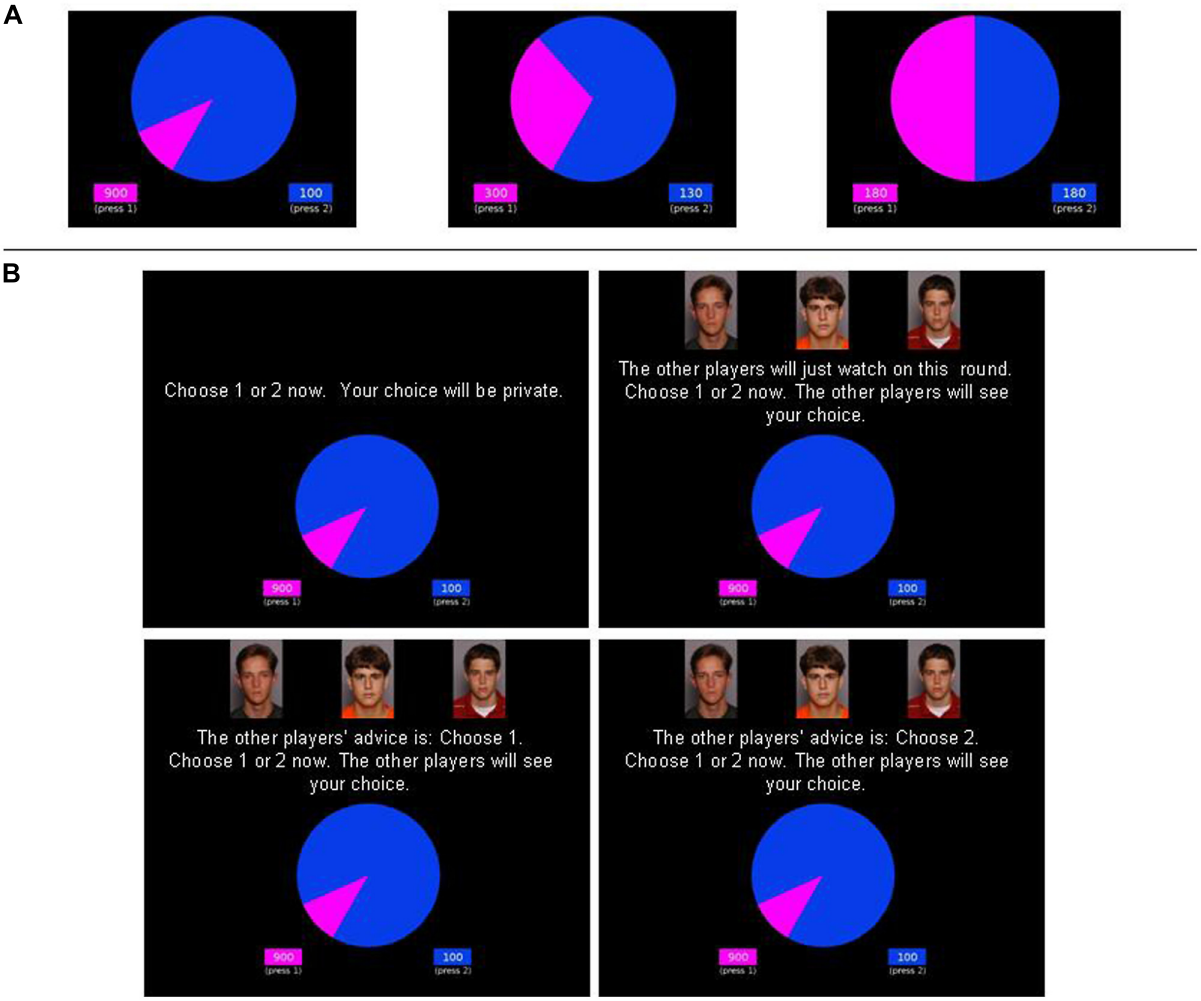
**Schematic diagram of the Wheel of Fortune task. (A)** There were three gamble types: high-risk gambles, medium-risk gambles, and even gambles. On each trial, gamble type was indicated by the relative sizes of the pink and blue wedges of the wheel. **(B)** Within each gamble type, there were four social conditions: Private, Observed, Advised Risky, and Advised Safe (shown for high-risk gambles only). Condition was indicated by the presence or absence of the peer photographs (age- and gender-matched to the participant) on the screen, and the text instructions. Note that the smaller, pink wedge of the wheel appeared on the left or right with equal probability. The combination of three gamble types and four conditions resulted in 12 trial types. Note that for the two Advised conditions, a previous text instruction (not shown) told participants to wait for the other players advice; this was shown for a random period between 5 and 10 s. Adult peer photographs were taken from the Radboud Faces Database (Langner et al., 2010). Adolescent peer photographs were taken from a set of headshots of adolescent actors posing different expressions (Guyer et al., 2009).

Adult participants were tested in the Department of Experimental Psychology and adolescent participants were tested in a quiet area of the school. After completing consent, participants were administered the Wheel of Fortune game (Ernst et al., 2004; Roy et al., 2011; Shad et al., 2011), a computerized, two-choice, decision-making task involving probabilistic outcomes. On each trial, participants saw a ‘wheel’ with two possible choices, represented as two ‘slices’ of differing size and color (see **Figure 1**). Slice size represented the probability of that color winning. After participants had chosen a color, the wheel was spun and landed on one of the colors. If participants had chosen the winning color, they won a given number of points (displayed next to the wheel). If participants chose the color that did not win, they won nothing.

There were 64 trials which were divided into three risk conditions (see **Figure 1**; **Table 1**): (a) *high-risk gambles* (24 trials), where choosing pink gave a 10% chance of winning 900 points and choosing blue gave a 90% chance of winning 100 points; (b) *medium-risk gambles* (24 trials), where choosing pink gave a 30% chance of winning 300 points and choosing blue gave participants a 70% chance of winning 130 points; and (c) *even gambles* (16 trials), where choosing pink or blue each gave a 50% chance of winning 180 points. Note that behavior on the high-risk gambles was our measure of risky decision-making, with the other two gamble types serving as baseline conditions. With these probabilities and reward values, the expected value for each of the two choices across trials (i.e., the chance of winning a certain number of points across blue versus pink choices) was always the same. Participants were told that the more points they won, the more money they would receive (but in fact, payments were not dependent on game performance).

**Table 1:** Proportion of trials on which participants chose the pink option (which was always the higher risk option on high-risk and medium-risk gambles).

		Age group			
		Adolescent		Adult	
		Mean	SD	Mean	SD
High-risk gambles	Private (six trials)	0.400	0.322	0.264	0.326
	Observed (six trials)	0.458	0.358	0.160	0.262
	Advised risky (six trials)	0.733	0.287	0.910	0.230
	Advised safe (six trials)	0.313	0.295	0.090	0.184

Medium-risk gambles	Private (six trials)	0.525	0.281	0.444	0.367
	Observed (six trials)	0.613	0.278	0.403	0.307
	Advised risky (six trials)	0.758	0.267	0.660	0.328
	Advised safe (six trials)	0.329	0.260	0.271	0.310

Even gambles	Private (four trials)	0.500	0.289	0.510	0.271
	Observed (four trials)	0.562	0.245	0.490	0.271
	Advised pink (four trials)	0.794	0.246	0.813	0.278
	Advised blue (four trials)	0.181	0.240	0.125	0.266

Participants played the game under four conditions (see **Figure 1**; 16 trials in each condition – see **Table 1**): (a) *Private,* where choices were private; (b) *Observed*, in which participants were merely observed by the (fictitious) peers; (c) *Advised Risky,* in which the peers advised the participant to choose the pink option (which was always the more risky option for high-risk and medium-risk gambles); and (d) *Advised Safe*, in which the peers advised the participant to choose the blue option (which was the less risky option for high-risk and medium-risk gambles). Peer advice was independent of outcome. For each of the four conditions there were six high-risk, six medium-risk, and four even gambles. The task was divided into two blocks: private (the 16 private trials) and peer (16 *Observed*, 16 *Advised Risky*, and 16 *Advised Safe* trials). Within each block, trials appeared in a different random order for each participant. Blocks order was counter-balanced between participants. The proportion of trials for which the participant chose the pink option (which was always the more risky option for high-risk and medium-risk gambles) for each condition (Private, Observed, Advised Risky, Advised Safe) and risk level (high, medium, even) formed the outcome variable.

On each trial, instructions appeared above the wheel, which made clear which trial type it was and, where relevant, what the peers’ advice was (see **Figure 1**). Before the game started, a digital photograph was taken of the participant and uploaded to the online game platform. Participants were told that the other players in the game were also taking part in the experiment at other locations around the country. The experimenter appeared to interact with the (fictitious) other experimenters who were administering the task to these other players via a fake instant messaging box. For the ‘peer’ blocks, the participant’s photograph was displayed on the screen at the beginning of the block alongside photographs of the three other ‘participants’ ostensibly taking part. The participant was then chosen, ostensibly at random, to play the game while the other players observed and advised. The other players’ photographs appeared on-screen throughout social blocks. During the Private block, no photos appeared on the screen.

After completing the task, adult participants were given post-study questionnaire which was used to assess whether they had believed that they were interacting with real other people. In particular, the questionnaire asked “How realistic did you think the game was as an online interaction?” with the experimenter following up with further questions to probe the degree to which the participant believed the deception. Data from 4 adult participants (two males and two females) indicated that they had not believed that they were interacting with real peers; their data was therefore excluded from all analyses (although repeating analysis that included these participants showed a broadly similar pattern of results to that reported below). Adult participants were then debriefed fully and paid for their participation. We were concerned that using the post-study questionnaire with the adolescent participants might raise doubts in their minds about the authenticity of the other participants and that this information would be likely to spread through the school, undermining the effectiveness of the deception with subsequent participants. We therefore asked participants to return to a separate debriefing session to complete the post-study questionnaire and learn more about the study once data collection in the school was complete. However, fewer than half of the participants came to the session, meaning that data on the effectiveness of the deception was unavailable for most of the adolescent participants. Moreover, scheduling difficulties meant that the delay between taking part in the study and completing the post-study questionnaire and debriefing session was up to 4 months for some participants. For this reason, we were not able to exclude adolescents who did not believe that they were interacting with real peers.

### DATA ANALYSIS

Data analyses were performed in SPSS version 20. An overall ANOVA with the factors risk, condition, age group, and gender was followed up with lower-level ANOVAs and then *t*-tests to examine the sources of the main effects and interactions. Significant interactions were followed up with *t*-tests. The Greenhouse-Geisser correction was used where the assumption of sphericity was violated. Because half of the participants completed Private trials before Peer trials (with the other half completing Peer trials first), Order was first included as a factor in analyses. As there was no significant main effect of Order; nor did it interact with Condition, it was not included in the analyses reported below. Moreover, initial analyses including gender showed that it was not a significant factor and so it was excluded from the analyses reported below.

## RESULTS

**Table 1** shows the proportion of trials on which participants chose the high-risk/pink option in each condition for each risk level.

A 4 (condition) × 3 (risk) × 2 (age group) mixed design ANOVA was performed on proportion of ‘risky’ decisions, revealing main effects of condition *F*_condition_(2.527,156.695) = 102.813, *p* < 0.001, risk *F*_risk_(1.795,111.296) = 4.861, *p* = 0.012, and age group *F*_agegroup_(1,62) = 10.296, *p* = 0.002. To explore the main effects of condition, pairwise comparisons of the four conditions (collapsed across age group and risk level) revealed that participants chose the risky option more often in the Advised Risky condition and less often in the Advised Safe condition than in either the Private or the Observed condition. To explore the main effects of risk, pairwise comparisons of the three risk levels (collapsed across age group and condition) showed that participants chose the risky option more often in the high risk than the medium or low risk conditions. Finally, to explore the main effect of age group, pairwise comparison of the two age groups (collapsed across all conditions and risk levels) showed that adolescents chose the risky option more often than the adults. However, these main effects were modified by a number of interactions: as well as significant age group-by-condition and condition-by-risk interactions, a significant 3-way interaction (age group-by-condition-by-risk) emerged, *F*(4.534,281.088) = 3.155, *p* = 0.011, partial η^2^ = 0.048. To decompose these effects, we examined the effects of age group and condition under each risk condition separately.

### HIGH-RISK GAMBLES

**FIGURE 2:**
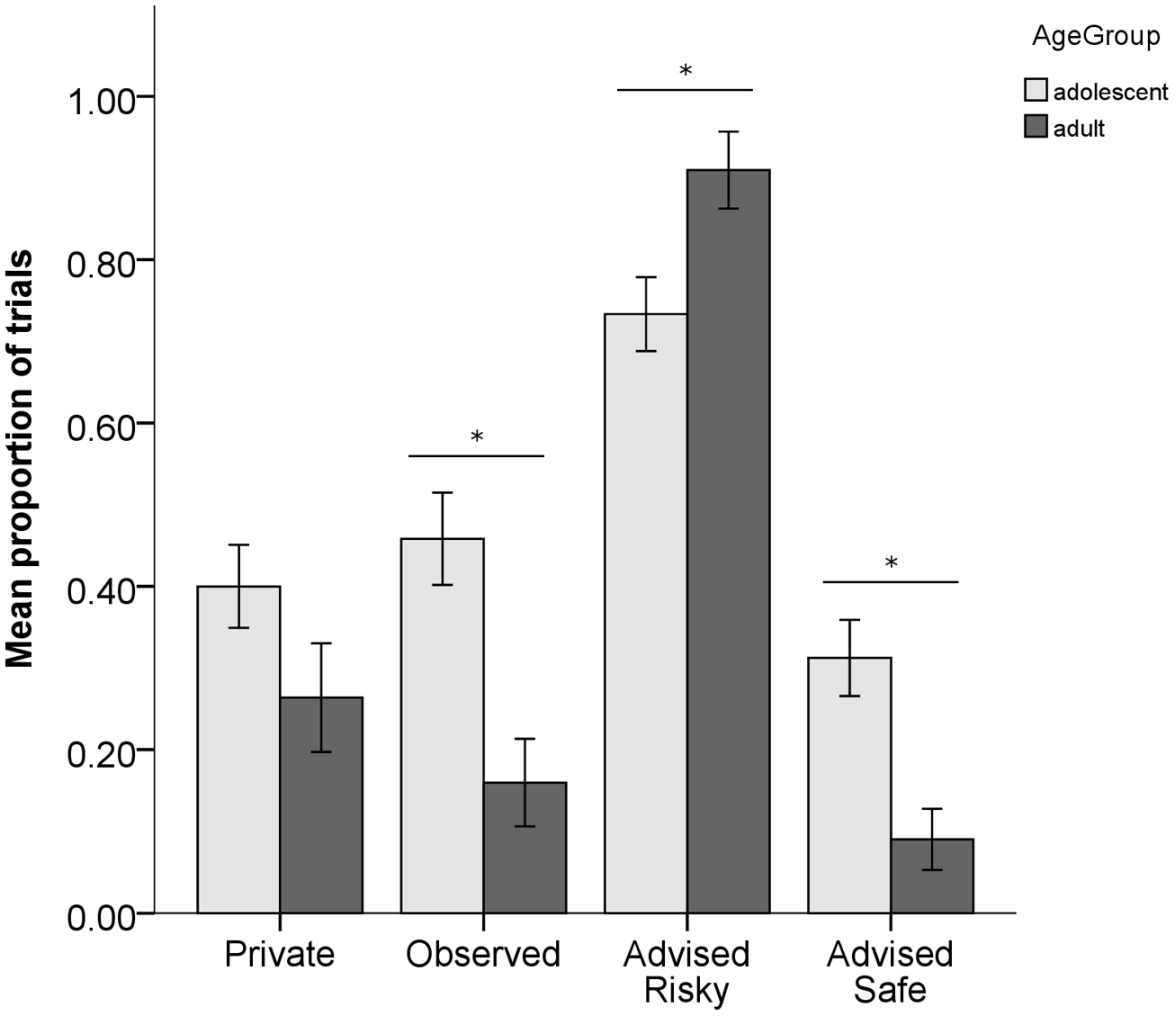
**Proportion of trials where participants chose the risky option for high risk gambles in each of the four conditions.** Light bars show adolescent data and dark bars show adult data. Data represent mean ± SEM; asterisks indicate significant differences between the groups.

For high-risk gambles, the 4 (condition) × 2 (age group) mixed design ANOVA, showed main effects of age group, *F*(1, 62) = 10.368, *p* = 0.002, partial η^2^ = 0.143, and condition, *F*(1.913,118.619) = 51.690, *p* < 0.001, partial η^2^ = 0.455. These effects arose because adolescents made significantly more risky decisions than adults across all conditions, and because across both age groups, participants tended to follow the advice, choosing the risky option more in the Advised Risky condition and less in the Advised Safe condition than in the Private or Observed conditions, which did not differ from each other. However, there was also a significant age group-by-condition interaction, *F*(1.913,118.619) = 7.361, *p* = 0.001, partial η^2^ = 0.106. Comparing the two age groups for each condition separately revealed no significant differences in behavior in the Private condition, *p* = 0.108; however, adolescents made more risky decisions than adults in both the Observed condition, *t*(59.363) = 3.835, *p* < 0.001, *d* = 0.95, and the Advised Safe condition, *t*(61.879) = 3.711, *p* < 0.001, *d* = 0.90. In contrast, adults made more risky decisions than adolescents in the Advised Risky condition, *t*(56.744) = 2.699, *p* = 0.009, *d* = 0.68 (see **Figure 2**).

### MEDIUM-RISK GAMBLES

For medium-risk gambles, only a main effect of condition emerged, *F*(3,186) = 35.548, *p* < 0.001, partial η^2^ = 0.364. Collapsed across age group, participants tended to follow the advice, choosing the risky option more in the Advised Risky than the Private condition, *M*_AdvisedRisky_ = 0.721, SD = 0.293; *M*_Private_ = 0.495, SD = 0.316; *t*(63) = 5.495, *p* < 0.001, *d*_z_ = 0.69, and conversely, chose the risky option less in the Advised Safe than the Private condition, *M*_AdvisedSafe_ = 0.307, SD = 0.279; *t*(63) = 5.116, *p* < 0.001, *d*_z_ = 0.64. The Observed versus Private conditions did not differ significantly. The main effect of age group and the age-group-by-condition interaction were not significant, *p* ≥ 0.060.

### EVEN GAMBLES

For even gambles, the only significant effect was a main effect of condition, *F*(3,186) = 61.278, *p* < 0.001, partial η^2^ = 0.497. This reflected the fact that participants tended to follow the advice in both Advice condition (whether this was advice for pink or for blue) relative to the Private condition, *M*_Private_ = 0.504, SD = 0.280; *M*_AdvisedPink_ = 0.800, SD = 0.257, *M*_AdvisedBlue_ = 0.160, SD = 0.249; Advised Pink vs. Private: *t*(63) = 6.378, *p* < 0.001, *d*_z_ = 0.80; Advised Blue vs. Private: *t*(63) = 7.251, *p* < 0.001, *d*_z_ = 0.91. Again, the Observed and Private conditions did not differ significantly. Neither the main effect of age group and the age-group-by-condition interaction was significant, *p* ≥ 0.452.

## DISCUSSION

Dramatic changes in risk-taking behavior occur in adolescence. Here, we focussed on how social context affects adolescents’ decisions about risky decisions and how this differs from adults. In a ‘virtual’ social environment, risk-taking behavior was successfully manipulated in all participants: choices in the two Advised conditions differed significantly from choices in the Private and Observed conditions. This was the case across all three risk levels. There was also a main effect of age suggesting that, consistent with prior studies, adolescents selected the risky option more. However, for the risky decisions only this main effect was modified by a number of other influences. First, when being merely observed by peers, adolescents took more risks than adults, despite comparable behavior in private. Second, whereas adults tended to follow safe advice from peers, adolescents tended to resist it. Third, although both adults and adolescents tended to follow risky advice from peers, under this condition, adults made more risky decisions than adolescents.

Compared with adults, adolescents made riskier decisions when being merely observed by peers. Importantly, this did not appear to be simply due to an overall tendency for adolescents to be more risk-prone, as there were no differences between adults and adolescents in the Private high-risk condition. Indeed, the finding was driven by the adults becoming *less* risk-seeking under observation than they were in private, whereas average behavior in the adolescents did not change. Thus when the stakes are high, peer observation is more likely to push adults to choose a safer course, whereas adolescents tend to persist in choosing the riskier option at the same rate. This finding resonates with previous work showing increased risk-taking behavior in adolescents compared with adults when peers are present (Gardner and Steinberg, 2005; Chein et al., 2011). However, our finding demonstrates that this can be driven by changes in adults’ behavior under observation – as well as that such shifts can occur even when peers are unknown to the participants and when they are merely present without saying anything.

Considering next the Advised Safe condition, during risky decision-making, adults tended to follow safe advice, but adolescents resisted, going against what their peers suggested significantly more than did adults. This finding underscores the fact that adolescents may be especially resistant to advice to follow a safer course of action – even when this advice comes from peers. Adolescents may have been more differentially sensitive than adults to the reward value of the points (which they believed could be redeemed for cash) over the social reward – peer approval or acceptance – which they might have gained by following peers’ advice. Another, perhaps related, possibility, is that adults and adolescents may have had different notions about peers’ motivations for advising the safe option. For example, adolescents may have been more likely to view the game as competitive rather than neutral or collaborative; this might have meant that some adolescents viewed ‘safe’ advice as being motivated by a Machiavellian attempt by peers to limit the number of points that the participant won. This finding is also interesting in light of evidence adolescents may sometimes resist peer influence to signal their independence as they develop their own sense of identity (Brechwald and Prinstein, 2011).

Our third finding of interest was that although both age groups tended to follow risky advice, with an overall increase in risky decisions in this condition compared to behavior in private, this pattern was more pronounced in adults than in the adolescents. This was unexpected and it is unclear why it occurred, although a number of possibilities can be suggested. For example, given that adults also followed ‘safe’ advice more than adolescents did, perhaps adults are more likely to simply follow *any* advice from new peers. However, one would then expect adults to *also* follow advice more than adolescents for non-risky decisions (i.e., medium-risk and even gambles) – which did not occur. Regardless of the reason, however, this finding highlights the need to consider the role of peer observation and peer advice separately when considering the effects of social context on risk-taking behavior – especially as the adults showed opposite shifts in behavior for the Observed condition and the Advised Risky condition.

A number of limitations must be acknowledged. First, concerns about the validity of adolescent post-study questionnaire data meant that we could not exclude adolescents who did not believe that they were interacting with real peers. Second, the task involved making choices about risks that were of known reward value and likelihood, which may not correspond well to real-world risks where the precise parameters are unknown or uncertain. However, the task is similar to other paradigms that are widely used to investigate risk-taking behavior (e.g., Ernst et al., 2004; Roy et al., 2011; Shad et al., 2011) and moreover, many interventions to reduce risk-taking behavior seek to do so by giving adolescents more accurate information about the risks of such behaviors. Our demonstration of peer influences on risk-taking behavior even with reward of known value and likelihood may explain why such interventions have would limited effectiveness in reducing adolescent – or indeed adult – risk-taking behavior. A final limitation is that the fictitious peers were strangers to the participants prior to taking part; familiar peers may have changed risk-taking behavior in different ways. This is particularly important because real-world adolescent risk-taking behavior more often takes place with friends rather than strangers or new acquaintances. Relatedly, we cannot exclude the possibility that our results in the Advised Risky and Advised Safe conditions may have been due to demand characteristics (e.g., participants may have thought that we expected or wanted them to follow peers’ advice and behaved accordingly). However, it seems likely that friends would produce more pronounced changes in risk-taking behavior – especially in adolescents, for whom peer relationships are especially salient. Because of this, our study represents a relatively conservative test for developmental differences in the effect of peers on risk-taking behavior. One strength of Gardner and Steinberg’s (2005) study was that they invited participants to bring friends into the lab to take part; peer observation/advice was therefore from individuals whom the participants knew well, making the findings more applicable to real-world contexts in which friends might influence young drivers’ risk-taking behavior. Future research with our paradigm could use a similar approach by leading participants to believe that they are interacting with real friends with whom they have come into the lab but this would involve having to control for strength/quality of the relationship with those friends across age-groups to ensure these variables did not confound peer influence effects. On the other hand, our findings could have important practical implications for contexts involving interactions with new or unfamiliar peers, including, for example, interactions via the internet. Our findings suggest that in such contexts, ‘safe’ advice from peers may be ineffective in reducing risk-taking behavior in adolescents. Further work to examine whether some unfamiliar peers are more influential than others (e.g., due to differential social status) in influencing adolescents toward safe behavior would be useful in this regard.

Despite these limitations, our data provide some preliminary findings on age-group differences in the role and influence of peers during risk-taking. These data should encourage future research in a number of areas. First, as our adult age range was relatively restricted with a relatively low mean (∼25), and as our adolescent sample covered a wide range of ages, future studies could recruit participants with a more continuous age range spanning late childhood to mid-to-late adulthood to investigate more subtle changes in peer influence on risk-taking that may occur across crucial developmental junctures. Future research could also examine the effects of same *vs.* opposite gender peers on risk-taking behavior. We found no effects of gender here; however, peers were always of the same gender as the participant, and there is some evidence for differential effects of male and female observers on risk-taking behavior (Simons-Morton et al., 2005), although same/opposite gender effects in adolescents have not been examined directly in an experimental paradigm. It may be particularly interesting to look at how effects of same/opposite gender peers change through adolescence as the possibility of romantic partnerships become more salient.

In summary, we used a novel experimental risk-taking task in virtual social contexts to demonstrate differences between adults and adolescents in risky decision-making behavior under observation and under advice for high-risk gambles. The strength of our paradigm is that it enables researchers to fully control the peers and advice that participants are exposed to, whilst retaining high ecological validity. Our findings suggest promising avenues for further work examining how interactions between biological, cognitive, and social factors produce such developmental differences in behavior, with possible implications for interventions to reduce risk-taking behavior in adolescence.

## Conflict of Interest Statement

The authors declare that the research was conducted in the absence of any commercial or financial relationships that could be construed as a potential conflict of interest.
